# Organically Modified Mesoporous Silica Nanoparticles against Bacterial Resistance

**DOI:** 10.1021/acs.chemmater.3c02192

**Published:** 2023-10-16

**Authors:** Montserrat Colilla, María Vallet-Regí

**Affiliations:** # Departamento de Química en Ciencias Farmacéuticas, Facultad de Farmacia, Universidad Complutense de Madrid, Instituto de Investigación Sanitaria Hospital 12 de Octubre i+12, Plaza Ramón y Cajal s/n, 28040 Madrid, Spain; † Centro de Investigación Biomédica en Red de Bioingeniería, Biomateriales y Nanomedicina (CIBER-BBN), Madrid 28029, Spain

## Abstract

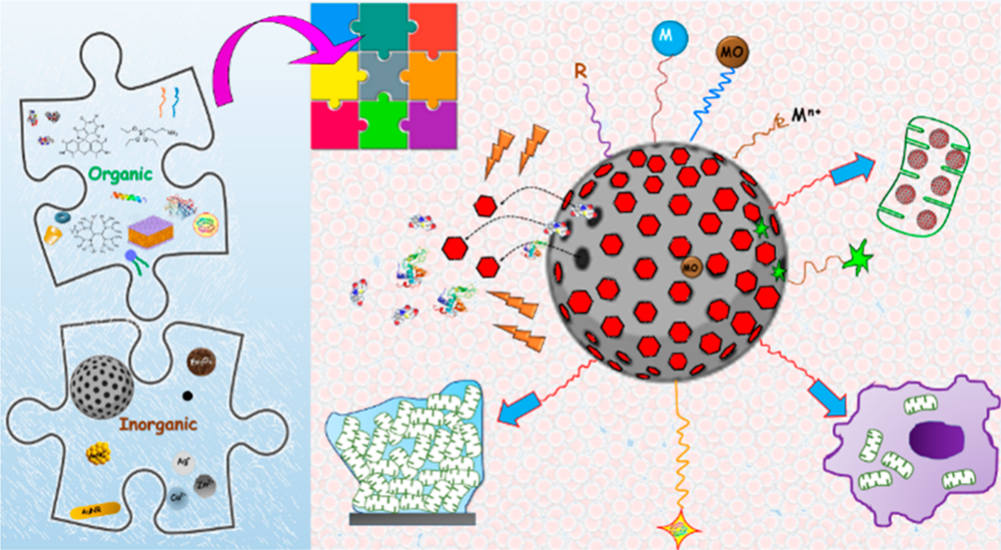

Bacterial antimicrobial resistance is posed to become a major hazard to global health in the 21st century. An aggravating issue is the stalled antibiotic research pipeline, which requires the development of new therapeutic strategies to combat antibiotic-resistant infections. Nanotechnology has entered into this scenario bringing up the opportunity to use nanocarriers capable of transporting and delivering antimicrobials to the target site, overcoming bacterial resistant barriers. Among them, mesoporous silica nanoparticles (MSNs) are receiving growing attention due to their unique features, including large drug loading capacity, biocompatibility, tunable pore sizes and volumes, and functionalizable silanol-rich surface. This perspective article outlines the recent research advances in the design and development of organically modified MSNs to fight bacterial infections. First, a brief introduction to the different mechanisms of bacterial resistance is presented. Then, we review the recent scientific approaches to engineer multifunctional MSNs conceived as an assembly of inorganic and organic building blocks, against bacterial resistance. These elements include specific ligands to target planktonic bacteria, intracellular bacteria, or bacterial biofilm; stimuli-responsive entities to prevent antimicrobial cargo release before arriving at the target; imaging agents for diagnosis; additional constituents for synergistic combination antimicrobial therapies; and aims to improve the therapeutic outcomes. Finally, this manuscript addresses the current challenges and future perspectives on this hot research area.

## 1 Introduction

Bacterial infections and induced diseases, such as sepsis, are the second-leading cause of death worldwide, with an estimated 13.7 million infection-related deaths in 2019.^1^ Bacterial antimicrobial resistance (AMR), which happens when modifications in bacteria provoke the drugs used to treat infections to become less effective, has emerged as a great hazard to global health in the 21st century. It is foreseen that AMR could kill 10 million people per year by 2050.^2^ A recent study estimated 4.95 million deaths associated with bacterial AMR in 2019, comprising 1.27 million deaths ascribed to bacterial AMR.^3^ Responsible for this disquieting data are common bacterial strains that develop multidrug resistance (MDR) when exposed to large amounts of or over a long time to the antibiotics used to treat and control bacterial infections.^4,5^ In this regard, the negative impact of coronavirus disease (COVID-19) on AMR should not be overruled, resulting from the inappropriate empirical use of antibiotics, in the context of lack of vaccines and effective drugs to treat this viral infection.^6,7^

Antibiotic resistance of bacterial infections is based on different mechanisms: the reduction of efflux transport, the modification of the target, the limitation of the drug uptake, and inactivation catalyzed by certain enzymes. Efflux pumps consist of certain protein pumps present in bacteria that can transport antibiotics from inside the cell to the outside. Bacteria have also developed resistance toward certain antibiotics thanks to a series of DNA mutations or even producing specific enzymes, which would end up in the target modification of that antibiotic. Additionally, bacteria also develop resistance thanks to some proteins that can bind to antibiotics or their targets, which would reduce the antibiotic uptake. Bacteria can also resist the action of antibiotics through their inactivation thanks to the action of self-produced enzymes that recognize and destroy those antibiotics. These mechanisms of resistance are divided in two groups: intrinsic, found across all strains, and acquired, appearing initially in a few strains and then spreading around. In this sense, acquired resistance presents a higher risk to human health.

In general, one of the main issues with antimicrobial resistance spread is the absence of fast diagnostic tools capable of identifying pathogens and detecting antimicrobial resistance. In fact, the identification of the resistance profile mainly depends on culturing that pathogen, which may delay the results for several days. This delay would contribute to a wrong application of the available antibiotics for viral infection, the use of the wrong antibiotics, or the overuse of broad-spectrum antibiotics. In this sense, and from a healthcare perspective, antibiotic resistance is responsible for more extended hospitalization of those patients that might suffer from an infection. On top of that, from a clinical perspective, antibiotic resistance could also affect the success rate of many other clinical procedures, such as chemotherapy or surgery.^8^

The current methods for the clinical diagnosis of bacterial infection are based on pathogen identification, using culture-dependent techniques, mass spectroscopy, or nucleic acid-based technology; or antibiotic susceptibility profiling, using phenotypic techniques or molecular techniques.

Most bacteria develop acquired MDR by exposure to conventional antibiotics due to their lack of selectivity toward pathogenic bacteria; troubles in reaching the target site of action; instability; poor solubility; low bioavailability; high doses; or dosage frequency needed to maintain therapeutic plasma concentrations. Moreover, the toxicity, side effects, poor patient compliance, and increased healthcare costs contribute. An aggravating factor is the absence of new classes of antibiotics in the pipeline, mainly due to the long, arduous and expensive path to antibiotic approval. The COVID-19 pandemic has also hampered progress, delayed clinical trials, and distracted attention the of the already limited investors.^9^ The current scenario claims for multidisciplinary scientific efforts to develop innovative strategies to combat antibiotic-resistant infections.

Nanotechnology has come into this landscape bringing up the chance to use nanoparticles (NPs) as effective nanocarriers capable of transporting and delivering antimicrobials to the target site,^10^ bypassing aspects associated with antibiotic bacterial resistance mechanisms (aggressive enzymes, cell wall permeability; MDR efflux pumps, alteration of pharmacological drug targets, intracellular bacteria and bacterial biofilms),^11,12^ and showing a high antimicrobial effect at low doses, thus minimizing toxicity and side effects.

The unique properties of nanomaterials have fueled their therapeutic and diagnostic potential applications to counter bacterial infections. Concretely, nanoscaled materials have been demonstrated to successfully deal with challenges associated with drug resistance and/or biofilm development. Among those materials, NPs have been used alone, e.g., silver NPs, because they can kill or inhibit the growth of bacteria. However, the clinical translation is those metal NPs have been hindered by their potential cytotoxicity, which has changed course toward the use of more biocompatible materials in the clinic, such as polymeric and lipid NPs, to transport antibiotics to fight bacterial resistance and enhance antibacterial activity. In this sense, different NPs have been explored to enhance the control delivery of different antibacterial agents, including organic NPs, such as liposomes, polymeric micelles, polymeric NPs, or solid lipid NPs; and inorganic NPs, including metallic NPs and mesoporous silica NPs.

The possibility to integrate organic and inorganic components into a unique nanomaterial opens a land of opportunities to tailor-made multifunctional nanosystems for a wide range of nanotechnology applications.^13^ Focusing on the development of drug delivery nanoformulations, this integrative approach has been demonstrated to overcome the limitations of independent constituents, such as poor stability, premature cargo leakage before reaching the target, low biocompatibility, poor storage stability, and intolerable toxicity.^14^ Hence, a wide variety of multicomponent nanosystems have been implemented for drug delivery, gene therapy, phototherapy, tissue regeneration, vaccines, biomolecule detection, cancer theranostics, and antibacterial therapy.^15−21^

Over the last 20 years, organically modified mesoporous silica nanoparticles (MSNs) have been extensively exploited as drug delivery systems for a wide range of biomedical applications,^22−33^ most recently including the treatment of bacterial infections.^34−40^ MSNs constitute excellent nanocarriers due to their unique features, including large loading capacity, biocompatibility, ease of manufacture, adjustable pore sizes and volumes, and high density of silanol groups on their surface, that could favor subsequent functionalization processes.^41,42^

**Figure 1:**
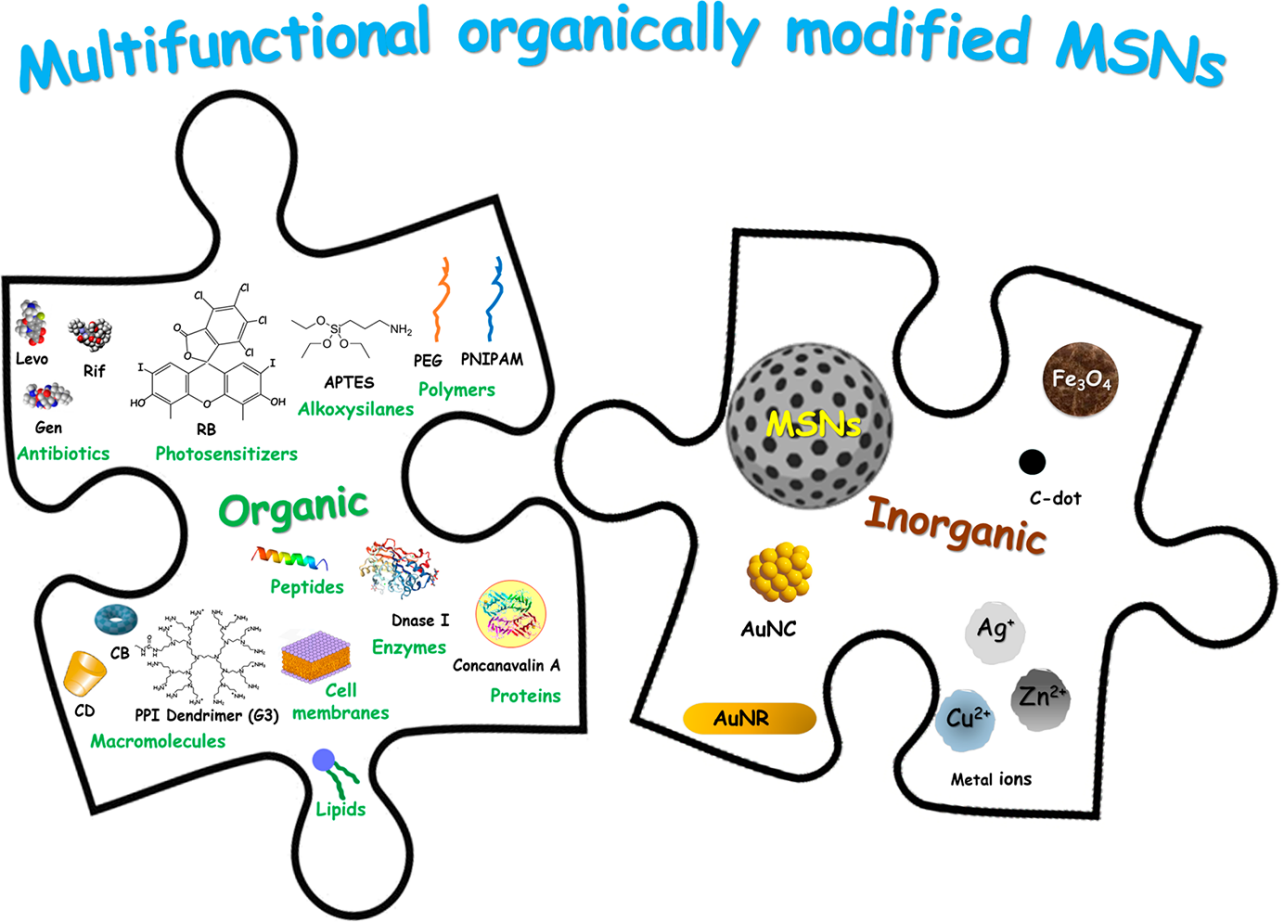
Typical organic and inorganic building blocks that compose multifunctional organically modified mesoporous silica nanoparticles (MSNs) for bacterial infection treatment. Abbreviations: Levofloxacin (Levo), Rifampicin (Rif), Gentamicin (Gen), Rose Bengal (RB), (3-aminopropyl)triethoxysilane (APTES), polyethylene glycol (PEG), poly(*N*-isopropylacrylamide) (PNIPAM), cyclodextrin (CD), curcubituril (CB), Poly(propyleneimine) dendrimers of the third generation (G3), carbon dot (C-dot), gold nanocluster (AuNC), gold nanorod (AuNR).

This perspective article focuses on organically modified MSNs against bacterial resistance. These multifunctional nanosystems are conceived as an assembly of inorganic and organic building blocks, each exhibiting distinct properties that determine its multifunctionality to evade bacterial defense mechanisms (Figure 1). Inorganic building blocks include MSNs as the principal assembly nanoplatform, metals (gold and iron oxide nanoparticles, and metal cations), and carbon dots (C-dots). Organic building blocks include polymers and copolymers, alkoxysilanes, lipids, isolated cell membranes, macromolecules, peptides, enzymes, proteins, photosensitizers, and antibiotics. By cleverly assembling these building blocks, almost limitless multifunctional MSNs can be designed to overcome the challenges associated with bacterial resistance. Herein, we present an up-to-date overview of the recent advances and contributions of the different multifunctional organically modified MSNs that have been developed to combat bacterial resistance. Initially this perspective article provides a brief overview on the different mechanisms of bacterial resistance. Thereafter, the innovative approaches developed so far to engineer advanced MSNs able to circumvent the different bacterial defense mechanisms are revised in detail. Finally, this manuscript addresses the current challenges and future prospects of this hot area of research.

## 2 Mechanisms of Bacterial Resistance

**Figure 2:**
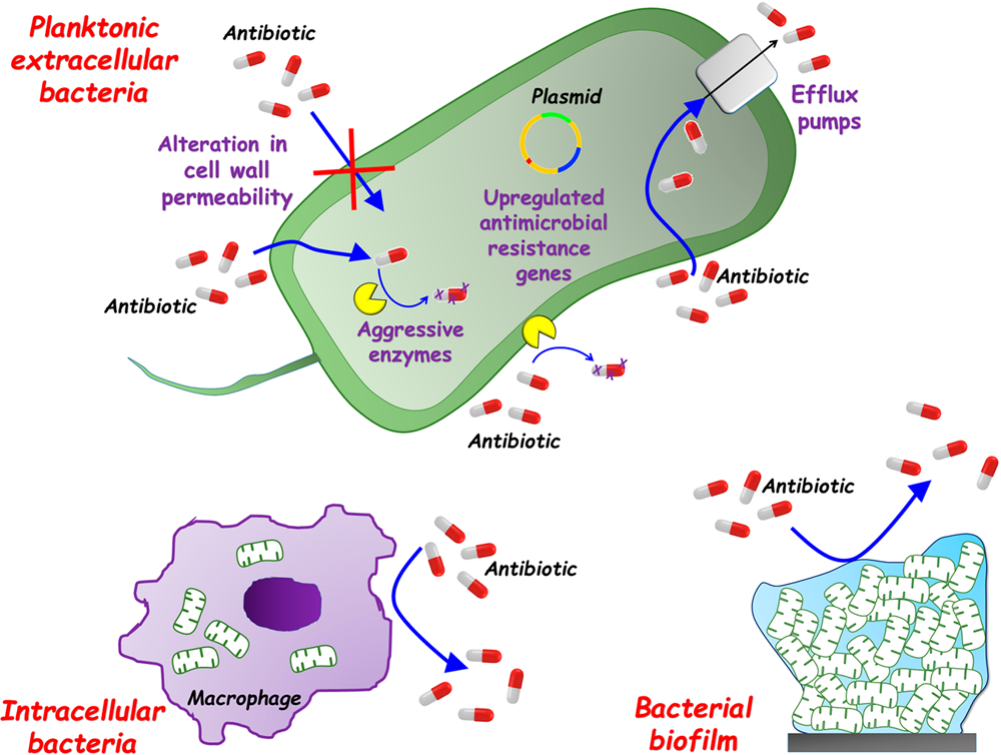
Schematic illustration of the main proposed mechanisms of bacterial resistance to antibiotics. The three types of resistant bacteria are shown.

The therapeutic action of conventional antibiotics is based on the inhibition of essential functions of bacteria, such as cell wall, protein, and nucleic acid synthesis and metabolic pathways.^3,4,43^ In this regard, bacteria have developed several protective mechanisms to defend against these actions.^11,12^ The main mechanism of bacterial resistance could be involved in a number of aspects, as illustrated in Figure 2 and discussed concisely below:

(i) *Aggressive enzymes*: bacteria can secrete various aggressive enzymes (e.g., hydrolases) capable of inactivating antibiotics, by modification, neutralization, or degradation, before reaching their targets. This is a key defense mechanism for bacteria.
(ii) *Alteration of cell wall permeability*: bacteria are capable of modifying the physical properties of their cell wall, altering its permeability and hindering the penetration of antibiotics inside the cell.
(iii) *Overexpression of multidrug resistant (MDR) efflux pumps*: upregulation of MDR efflux systems to pump antibiotics out of the bacteria and decrease the intracellular drug concentration. This protective mechanism is a fundamental impediment to antibiotic accumulation in bacteria.
(iv) *Upregulated antimicrobial resistance genes*: bacteria can rearrange the genetic code of antibiotic targets, such as certain proteins, to increase persistence and decrease susceptibility.
(v) *Intracellular infection*: some pathogenic bacteria, such as *Staphylococcus aureus* (*S. aureus*), *Mycobacterium tuberculosis* (*M. tuberculosis*), *Salmonella*, and *Listeria* are able to settle in specialized phagocytic cells, in particular macrophages, which not only protect them from eradication by the host immune system, but also from antibacterial agents. Over extended time periods, intracellular bacteria behave as a “Trojan horse”, causing recurrent infections, as they have evolved mechanisms to manipulate host membrane trafficking, remodel bacteria-containing vacuoles, modulate cell death signaling, and increase the longevity of the replicative compartment in order to survive and multiply therein.^44^ Intracellular bacterial infections are difficult to treat due to the inability of traditional antibiotics to penetrate, accumulate, or be retained in mammalian cells.
(vi) *Bacterial biofilms*: Up to 80% of chronic and recurrent infections are due to bacterial biofilms.^45^ Biofilms are organized surface-associated bacterial colonies enclosed in a matrix of self-secreted extracellular polymeric substances (EPSs)^46−49^ The EPS matrix essentially consists of polysaccharides, proteins, lipids, and extracellular DNA. Contrarily to free-floating planktonic bacteria, the EPS matrix creates a singular local microenvironment that enables cell-to-cell interactions, enhancing resource uptake, surface adhesion, and digestive capacity, while inhibiting bacterial dehydration and providing protection from the immune system and external agents (e.g., antibiotics).^50^ Using these activated facets, the EPS matrix can not only hinder the penetration of antibiotics into the biofilm but also concentrate bacterial cell products capable of degrading drugs and driving phenotypic differentiation. Finally, the heterogeneity of the biofilm produces gradients of nutrients and bacterial metabolites, resulting in regions where bacteria remain dormant. These dormant cells are highly resistant to antibiotics, which typically target growing and metabolically active bacteria.^51^ As a result, biofilm bacteria have shown 10 to 1000 times more resistance to antibiotics than planktonic bacteria.^45,52,53^

## 3 Multifunctional MSNs against Bacterial Resistance

Nanoparticles able to transport antibacterial agents could defeat the antibiotic resistant barrier thanks to their capacity of protecting those agents against hydrolysis, increasing the uptake into bacteria, and circumventing the bacterial efflux pump. As it has been highlighted throughout this review, mesoporous silica nanoparticles have been extensively investigated as nanocarriers of antibiotics because they can improve the delivery of those antibiotics to bacteria. Furthermore, MSNs could be doped with different metal NPs, metal oxide NPs, or metal ions to increase the antibacterial effect.

The mechanism of MSNs to combat bacterial resistance is based on the fact that those nanocarriers are able to transport large quantities of therapeutic agents into bacteria. The use of those nanocarriers to transport antibiotics offers many advantages, such as protection of the cargo during the journey, a great control of the antibiotics release kinetics, and the possibility of engineering triggered release to specific stimuli. The antibiotic release mechanism can produce a sustained release of the cargo, which might provide a long lasting antimicrobial efficacy and ensure a pronounced exposure of bacteria to a greater local concentration of the drug while overcoming many potential side effects. Additionally, MSNs might display enhanced membrane permeability thanks to the possibility of engineering their surface. In this sense, MSNs can be organically modified at their surface to adhere to the surface of bacteria through different mechanisms, such as electrostatic interactions either between the positively charged peptidoglycans present in Gram-positive bacteria walls and the negatively charged unmodified MSNs, or between the negative charged phospholipids from bacterial cell-wall and positively surface of amine-modified MSNs; hydrophobic forces between the phospholipids rich bacterial cell-wall and the hydrophobic surface of engineered MSNs; or ligand–receptor interactions between specific membrane receptors overexpressed in the bacterial cell-wall and specially selected targeting agents grafted on the surface of MSNs. Those mechanisms would guarantee a great accumulation of MSNs loaded with large therapeutic loads at the outer surface of bacteria. This might be of great importance, because it will help those antibiotics to cross bacterial walls and membranes and entering bacterial cytoplasm to fight them. To achieve this, MSNs are normally internalized through endocytosis, thanks to their encapsulation into endosomes and lysosomes. Thanks to the specifically designed external functionalization of MSNs to show buffering capacity, they reduce the acidic environment of those endosomes and lysosomes. The bacteria cells would then influx chloride ions along with water to equilibrate that proton removal. As a consequence, both endo- or lysosomes would swell due to the enormous amount of water molecules introduced and, eventually, those vesicles would be disrupted leading to the subsequent particle release into the cytoplasm of the bacteria cells. Then, the antibiotic agents would be safely released into the cytoplasm of the bacteria accomplishing the mission of the nanocarrier.

Metal NPs inhibit the growth or even kill bacteria through the inhibition of the synthesis of the bacterial cell-wall, through their interference in the protein expression process, or even through the damage of bacterial DNA.^54^ Therefore, the combination of metal nanoparticles or metal cations with MSNs can improve their performance against bacterial infection. Different approaches include Ag^+^ ions released from MSNs that can interact with subcellular organelles of pathogenic microorganisms and generate Reactive Oxygen Species (ROS) in the proximity of bacteria.^55^ Similarly, copper containing MSNs have shown potent antibacterial properties thanks to the oxidative stress generated by the presence of ROS.^56^ In general, the introduction of metal ions into the framework of MSNs can contribute to improve certain drug delivery properties, such as a better control over the antibiotics release or the surface electrical charge of the nanocarriers.^57^

**Figure 3:**
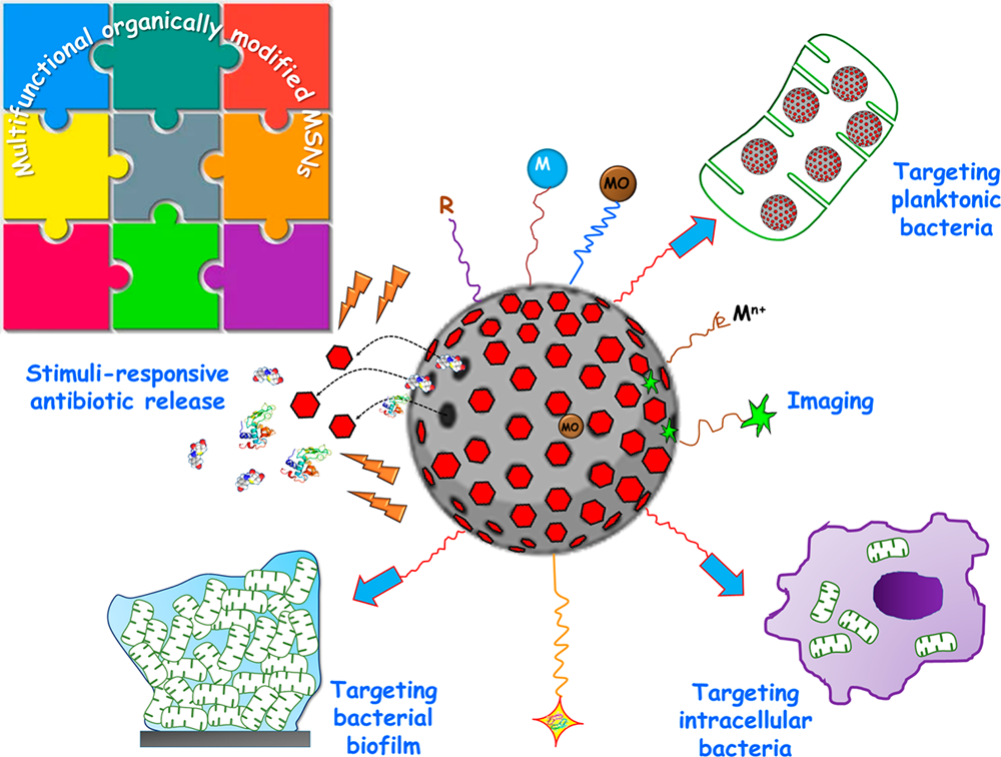
Assembly of organic and inorganic nanoscale building blocks to construct multifunctional MSNs against bacterial resistance. Ligands targeting planktonic bacteria, intracellular bacteria, or bacterial biofilm (blue arrows) can be incorporated on the outermost surface. Antibiotics and/or antibiofilm agents (proteins, enzymes, and peptides) can be loaded into the mesopores, and then stimuli-responsive nanogates (red nanocaps) can be incorporated to block the mesopores and prevent leakage of the therapeutic payload before reaching the target. Upon exposure to internal (endogenous) or external (exogenous) stimuli (orange rays), pore uncapping and payload release occurs. Antimicrobial metal nanoparticles (M), metal oxides (MO), and cations (M^n+^) can be integrated into the mesoporous structure or anchored to the external surface of MSNs. Biocompatible hydrophilic polymers (in orange), such as PEG, can decorate the outer surface to produce “stealthy” nanosystems. Decorating the outer surface with different organic functions (R) allows tailoring the surface charge. Finally, molecular imaging probes (green stars) can be embedded in the mesoporous matrix or grafted onto MSNs.

The manufacture of organically modified MSNs involves myriad interactions between the organic–inorganic components, whether covalent, noncovalent, or a combination of both. Combining different organic and inorganic building blocks in MSNs nanoplatforms allows for multifunctional nanocarriers with enhanced biological characteristic that can enhance therapeutic efficacy and reduce and/or overcome antibiotic resistance. Figure 3 shows different possibilities for assembling organic and inorganic building blocks to construct multifunctional MSNs against bacterial resistance. These modular components include targeting agents for selective transport of antimicrobials to the site of infection; stimuli-responsive nanogates to prevent premature release of therapeutic payload; imaging agents; and additional elements that enable the development of synergistic combinations of antibiotic delivery with other therapeutic strategies (e.g., photodynamic therapy, PDT, photothermal therapy, PTT, etc.) for synergistic antibacterial activities, as it will be detailed in the following sections.

### 3.1 Targeted Organically Modified MSNs

Targeted antimicrobial delivery aims to accumulate the drug at the target site, which enhances the therapeutic effect to reduce doses and dosing frequency and thereby reduces side effects. Thus, improving the efficiency of drug delivery inside the cell slows down the development of bacterial AMR. The assembly of targeting ligands on the outer surface of MSNs produces multifunctional nanosystems that not only specifically interact with the target (planktonic bacteria, intracellular bacteria, or bacterial biofilms), but also activate additional mechanisms of action attributed to the nanocarrier itself, such as destabilization of the bacterial cell wall or increased penetrability of the biofilm.^40^ This section discusses recent scientific efforts to design targeted organically modified MSNs to combat bacterial resistance.

#### 3.1.1 Targeting Extracellular Bacteria

The goal of targeting extracellular bacteria is to circumvent the defense mechanisms of isolated free-living planktonic bacteria by enhancing the uptake and intracellular concentration of antibiotics. Different approaches have been developed to achieve this goal.

**Figure 4:**
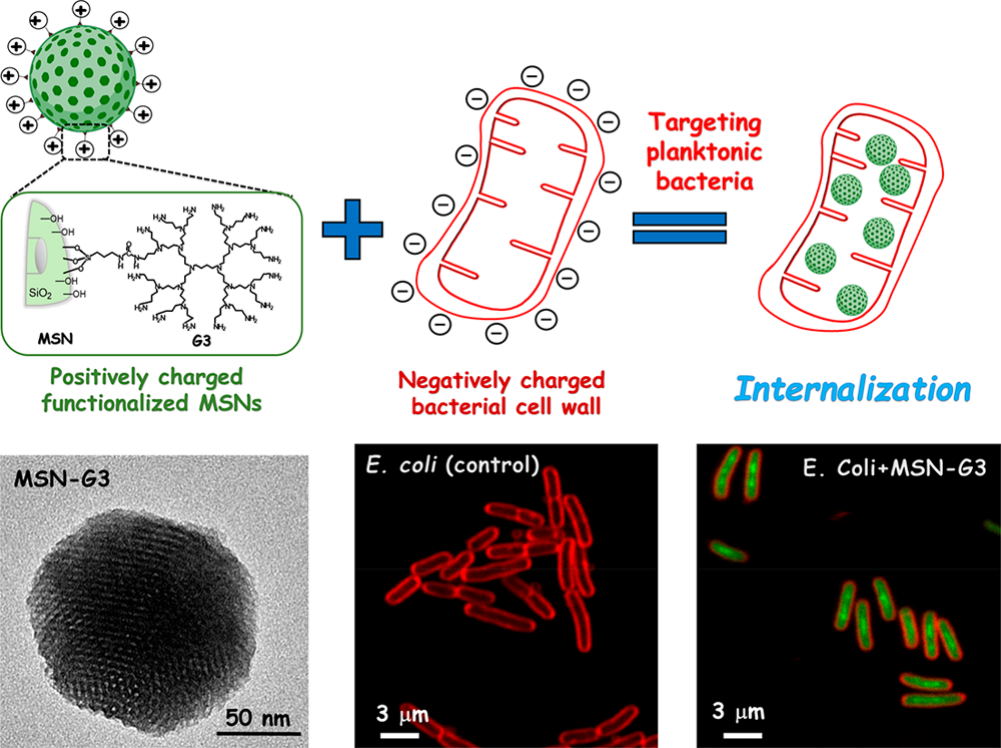
Schematic representation of the method described by González et al. for targeting organic modified MSNs to planktonic *E. coli* bacteria.^61^ Top: positively charged organic–inorganic hybrid mesoporous nanosystem (MSN-G3) composed by MSNs and the poly(propyleneimine) (PPI) dendrimer of the third generation (G3) covalently anchored to the external silica surface. The electrostatic attraction interaction between the positively charged MSN-G3 and the negatively charged Gram– *E. coli* bacterial cell wall triggers cell membrane disruption and internalization of the nanosystem. Bottom: transmission electron microscopy (TEM) image of MSN-G3 nanosystem (left); confocal microscopy images of planktonic *E. coli* control culture (center), where the *E. coli* cell membrane was stained in red using FM4-64FX; and *E. coli* culture after 90 min of incubation with 10 mg mL^–1^ of MSN-G3 (right), where MSNs were tagged in green during the synthesis process using fluorescein. Adapted with permission from ref (61). Copyright 2018 Elsevier.

Surface charge is the main factor affecting the interaction between NPs and bacteria, due to the negative charge of bacteria cell walls.^58^ Positively charged NPs can not only electrostatically attach and accumulate on the cell wall of Gram-positive (Gram+) and Gram-negative (Gram−) bacteria, but also disrupt metabolic pathways, perforate, or cause membrane leakage.^59,60^ Using this approach, González et al. covalently attached a polycationic dendrimer, poly(propyleneimine) dendrimer of third generation (G3), to the external surface of MSNs to enhance *E. coli* cell wall permeation and internalization of the nanosystem (Figure 4).^61^ Thus, the subsequent loading of levofloxacin into the nanosystem allowed the delivery of large amounts of antibiotics inside the bacteria,^61^ whereas the transport of some bactericidal metal ions such as Zn^2+^ and Ag^+^ produced synergistic antimicrobial effects.^62^ In another study, polyamine-decorated MSNs were proved to cause cell membrane disruption in Gram+ *Listeria monocytogenes*, showing a hundredfold higher antimicrobial effect than free polyamines.^63^ Martínez-Máñez and co-workers used the cationic polymer poly-l-lysine (ε-pLys) as a dual capping and targeting agent on antimicrobial-loaded MSNs. The positively charged lysine residues damaged the bacterial cell wall and allowed efficient delivery inside the bacteria.^64,65^ In another work, Alsaiari et al. developed innovative organically modified MSNs incorporating several functional elements, most notably cationic lysozyme to detect and inhibit the growth of Gram– *E. coli* and Gram+ *B. safensis* bacteria.^66^

The ligand–receptor binding concept has also been applied to the design of MSNs decorated with ligands that specifically bind to surface receptors overexpressed on the cell wall of planktonic cells to enhance the antibacterial effect by improving antibiotic uptake or overcoming bacterial MDR related with the efflux pump system. These targeting ligands include antibodies,^67,68^ aptamers,^69^ sugars,^70,71^ folic acid,^72^ and vancomycin,^73^ among others.

The use of biomimetic approaches inspired by nature, such as decorating the outermost surface of MSNs with bacterial outer membrane vesicles (OMV)^74^ or virus-like coatings,^75,76^ produces camouflaged hybrid MSNs with bacterial-like characteristics. This similarity increases the affinity of bacteria for biomimetic NPs and leads to higher uptake rates.

#### 3.1.2 Targeting Intracellular Bacteria

As mentioned above, many bacterial infectious diseases are caused by facultative pathogens capable of surviving in phagocytic cells.^77^ The intracellular localization of these bacteria protects them from the host defense mechanisms and from some antibiotics with poor penetrating ability into phagocytic cells. This section overviews the recent advances in organically modified MSNs for targeted delivery of antibiotics directly into the intracellular infection microenvironment.

In a first approach, Zink and co-workers developed MSNs equipped with a polyethylenimine (PEI) polymer to release rifampicin into *M. tuberculosis*-infected macrophages.^78^ The PEI polymer was immobilized on MSNs by electrostatic interaction with grafted phosphonate groups, leaving the empty mesoporous cavities available for antibiotic loading. PEI provided the nanosystem with a positive charge, enhancing uptake of MSNs by human macrophages, trafficking to acidified endosomes, and facilitating the release of high concentrations of drug intracellularly to kill *M. tuberculosis*.

**Figure 5:**
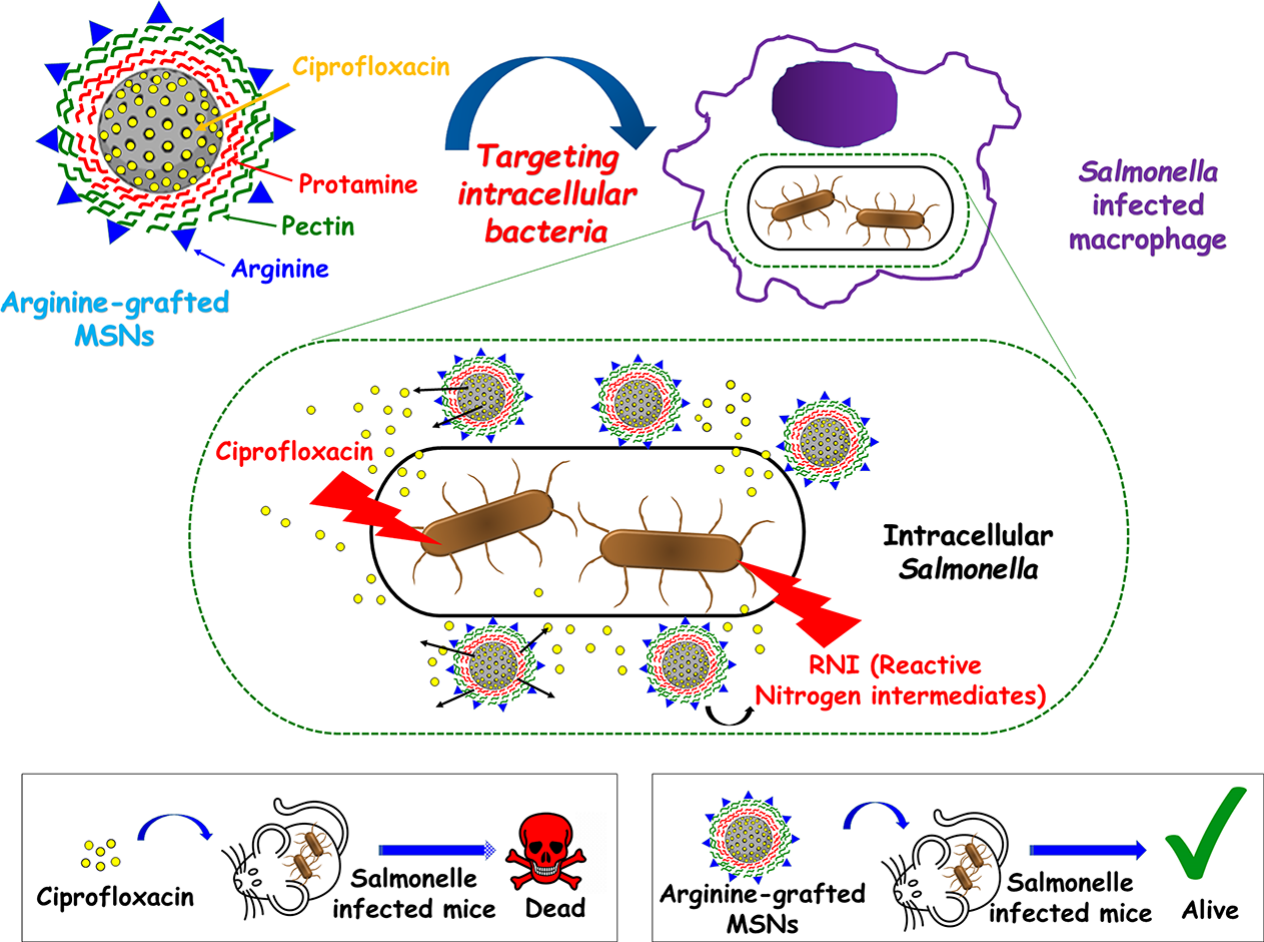
Schematic illustration of the method reported by Mudakavi et al. for targeting organically modified MSNs to intracellular *Salmonella* bacteria.^80^ Arginine-grafted MSNs target intracellular *Salmonella* to deliver ciprofloxacin into the intracellular niche. The effect of reactive nitrogen intermediates (RNI) and the colocalization of the MSNs with the intracellular *Salmonella* containing vacuole results in a successful antibacterial effect *in vivo*. Adapted with permission under a Creative Commons CC-BY 3.0 from ref (80). Copyright 2017 The Royal Society of Chemistry.

Another strategy is to use small targeting ligands, such as certain amino acids, whose receptors are upregulated in *Mycobacterium-*infected cells. For example, *Salmonella* infections have been reported to increase Arginine (Arg) uptake in the infected host cell.^79^ Thus, Mudakavi et al. developed protamine and pectin-coated, Arg-decorated MSNs to treat intracellular *Salmonella* with ciprofloxacin (Figure 5).^80^ The increased antibacterial activity compared to free ciprofloxacin is derived from colocalization of the nanosystem with intravacuolar *Salmonella* and the localized release of the antibiotic. In addition, the coordinated effect of enhanced antibiotic release, intracellular targeting, and reactive nitrogen species production resulted in enhanced antibacterial activity.

Antimicrobial peptides (AMP) with affinity for certain pathogenic bacteria have also been used to combat intracellular infections.^81,82^ Yang et al. decorated the outermost surface of lipid bilayer-coated MSNs with the synthetic cationic AMP ubiquidin (UBI)_29–41_, which exhibits high binding affinity for the anionic bacterial cell wall, to target *S. aureus*-infected preosteoblasts and macrophages.^81^ Lipid bilayer coating and UBI_29–41_ modification of gentamicin-loaded MSNs enhanced internalization in mammalian cells and showed excellent targeting and antimicrobial efficacy against intracellular *S. aureus* both *in vitro* and *in vivo*. In another work Rathnayake et al. developed AMP (LL-37)-targeted MSNs as colistin delivery systems to treat mammalian lung epithelial cells infected with *Pseudomonas aeruginosa*.^82^ LL-37 is an amphiphilic peptide that recognizes the outer membrane of Gram– *P. aeruginosa*. A 6.7-fold increase in the antimicrobial efficacy of colistin encapsulated in the LL-37 targeted nanosystem was observed compared to the free antibiotic. Finally, successful targeted inhibition of intracellular bacteria within lung epithelial cells was demonstrated, as only 7% bacterial viability was determined after treating infected-mammalian cells with the complete nanosystem.

#### 3.1.3 Targeting Bacterial Biofilm

Biofilms are based on a community of microorganisms that are irreversibly attached to a surface and embedded in a polysaccharide matrix. This self-produced matrix protects bacteria against antibiotics and the host immune system. The resistance to antimicrobial agents is mainly based on the physical hindrance of the matrix, whose shielding capacity can be increased by the presence of bacterial and host DNA together with certain proteins. Additionally, the matrix might contain certain enzymes capable of degrading antimicrobials, and more importantly, there might be some efflux pumps that also reduce the antimicrobials action. The process of biofilm formation can be described in four consecutive steps, which is (1) bacterial adhesion; (2) bacterial growth in different layers; (3) bacterial maturation; and (4) final biofilm formation. Additionally, biofilm can detach and disseminate into other tissues for further colonization.

The biofilm itself is a highly hydrated and chemically complex matrix that can store many nutrients together with other microbes or noncellular components, such as inorganic minerals and crystals.^83^

Although certain bacterial biofilms may be beneficial due to their protective role in, for example, gut epithelial cells to create a barrier against pathogens, in the clinical context they are generally considered as an important source of bacterial pathogens for patients. They are typically the cause of chronic, nosocomial and medical-device infections. Regarding the types of biofilms, and although both Gram-positive and Gram-negative bacteria are able to develop biofilms on medical devices, the most common types of biofilms encountered in clinical settings are *Enterococcus faecalis*, *Staphylococcus aureus*, *Staphylococcus epidermis*, *Streptococcus Viridans*, *E. coli*, *Klebsiella pneumoniae*, *Proteus mirabilis*, and *Pseudomonas aeruginosa*.^84^ From all of them, the most frequent biofilms found in clinical settings are *S. aureus* and *S. epidermis*, which are estimated to be responsible of about 40–50% of prosthetic heart valve infections, 50–70% of biofilm infections found in catheters, and 87% of infections in the bloodstream.^85^

Among the different approaches explored for biofilm eradication, several antibiotics substitutes have been explored, such as quorum-sensing inhibitors, bacteriophages, enzymes, surfactants, or selected antimicrobial probes.^86^ However, several disadvantages have fueled the search for different approaches, such as those found in nanotechnology.

Current nanotechnology-based approaches to efficiently control and/or eradicate biofilm-related infections focus on the design of advanced nanocarriers that target biofilm, destroy EPS, and enhance the biofilm permeability of antimicrobial substances.^86^

There are different nanocarriers that have been explored to combat biofilm infection, such as polymeric NPs, liposomes, lipid NPs, polymeric micelles, and magnetic NPs. And based on their intrinsic properties, each type of NPs presents some benefits or disadvantages. However, most of this research has been carried out *in vitro*, with very few of them *in vivo*. From the clinical perspective, there are only a couple of clinical trials: Arikace, a liposomal formulation of amikacin for inhalation, and Fluidosomes, a liposomal formulation of tobramycin. The reasons for this lack of clinical translation might be found in the intrinsic nanocarriers limitations, the lack of knowledge about the antibiofilm mechanism of nanomedicines, and the manufacturing and large-scale production of nanocarriers that were originally designed and created in small batches in the lab.

**Figure 6:**
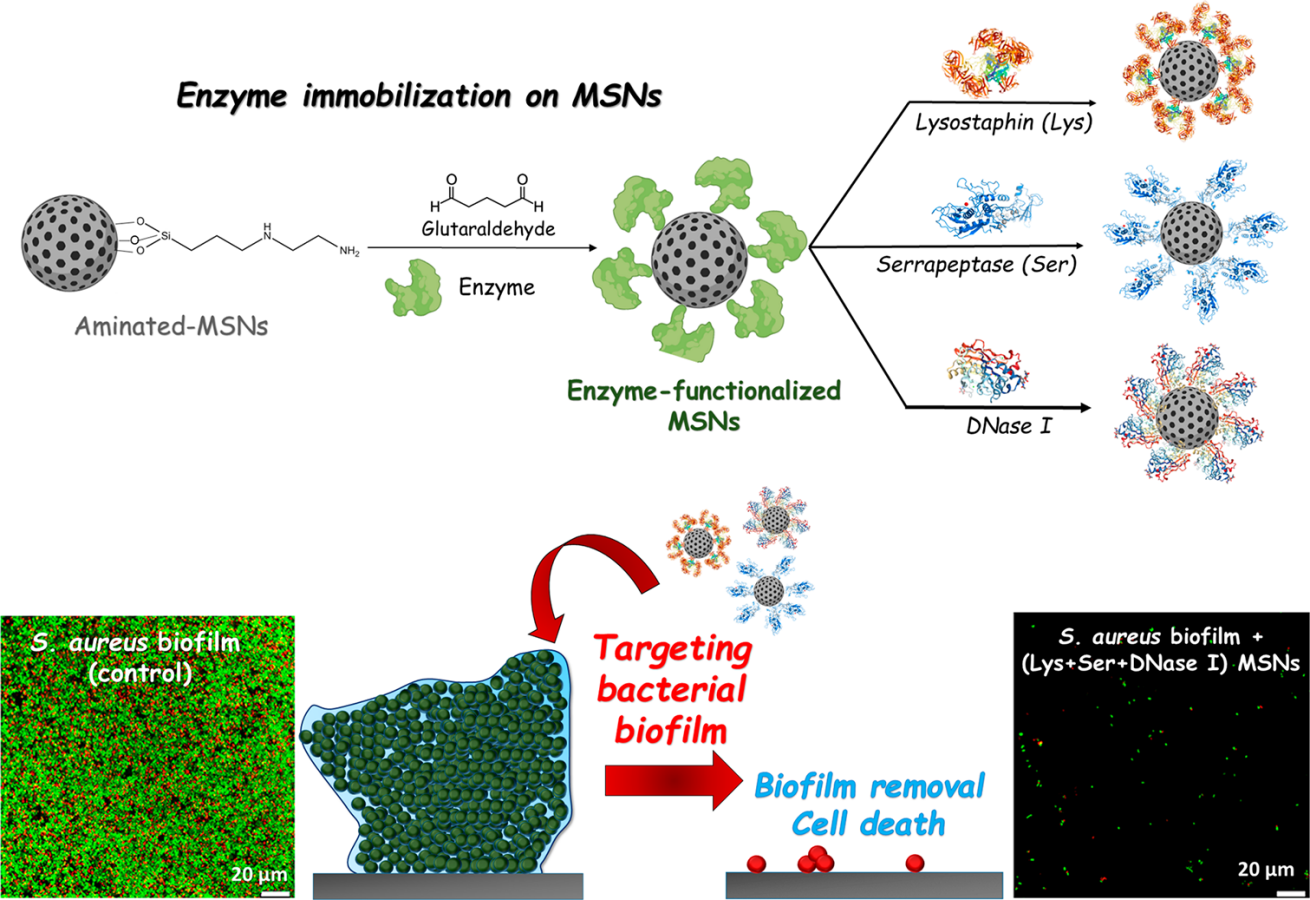
Schematic description illustrating the strategy reported by Devlin et al. to design enzyme functionalized MSNs to target *S. aureus* bacterial biofilm.^89^ Top: Synthetic procedure for the independent immobilization of three enzymes (lysostaphin, serrapeptase, and DNase I) on aminated-MSNs to produce enzyme-functionalized MSNs. Bottom: Representation of the proposed effect of MSNs functionalized with enzymes in *S. aureus* biofilm, leading to the removal of biofilm and cell death. Confocal laser scanning microscopy images of methicillin-resistant *S. aureus* (MRSA) biofilms before (control, left) and after exposure to 0.33 mg mL^–1^ (Lys + Ser + DNase I) MSNs (right). Live bacterial cells (green) were stained using SYTO 9 whereas dead cells (red) were stained with propidium iodide. Adapted with permission under a Creative Commons CC-BY-NC 4.0 from ref (89). Copyright 2021 Dove Medical Press Ltd.

Among the different nanocarriers, organically modified MSNs own excellent properties to load, protect, and release biofilm matrix-degrading agents, such as certain enzymes, e.g., lysozyme^87^ or DNase I,^88^ that reduce EPS cohesiveness and enhance antibiofilm efficacy. Another approach was to decorate the outer surface of MSNs with enzymes that can target bacterial biofilms, producing the biofilm matrix’s dispersal and bacterial cell death. Thus, Devlin et al. individually immobilized three different enzymes, lysostaphin (Lys), serrapeptase (Ser), and DNase I, on the surface of MSNs (Figure 6).^89^ This study showed that the combination of the three enzyme-modified nanosystems led to the near-complete eradication of methicillin resistant (MRSA) *S. aureus* biofilms, EPS dispersal, and significant decrease in cell viability.

**Figure 7:**
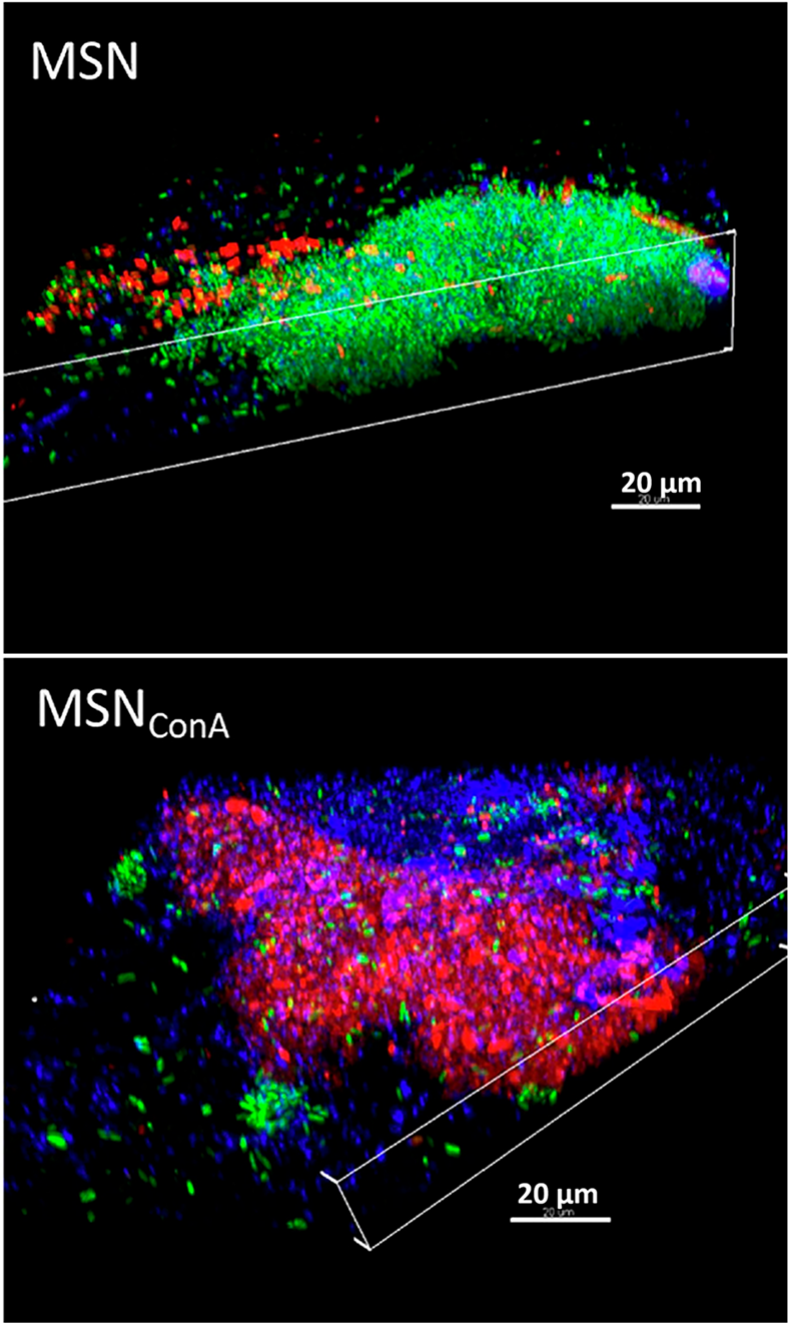
Confocal microscopy study of the internalization of red-labeled pristine MSN and MSN_ConA_ in preformed *E. coli* biofilms after 90 min of incubation with 50 μg mL^–1^ of NPs.^91^ 3D reconstruction shows that MSNs are localized onto the biofilm surface, whereas MSN_ConA_ penetrate the biofilm and are located at different depth levels. Live bacteria are stained in green (SYTO), nanoparticles in red (RhB), and the EPS biofilm matrix in blue (calcofluor). Reprinted with permission from ref (91). Copyright 2019 Elsevier.

Active targeting can be achieved by decorating MSNs with specific ligands to biofilm receptors. For example, lectins, such as concanavalin A (ConA), can bind glycans with high specificity.^90^ Martínez-Carmona et al. developed MSNs decorated with ConA (MSN_ConA_) and loaded with levofloxacin.^91^ ConA was used to target MSNs toward glycans present in the EPS biofilm matrix, allowing efficient penetration into *E. coli* biofilm (Figure 7) and increasing the antimicrobial effect of the antibiotic. Aguilera-Correa et al. used Arabic gum (AG) polysaccharide as the targeting ligand to coat MSNs.^92^ The AG-decorated MSNs showed high affinity for *E. coli* biofilms and remarkable antibacterial power thanks to the bactericidal effect of the moxifloxacin loaded in MSNs, and the disaggregating effect of the colistin embedded in the AG coating. The nanosystem eliminated more than 90% of the bacterial load on infected bone in a rabbit model of implant-associated osteomyelitis caused by *E. coli*. Recently, Moradi et al. conjugated MSNs with a novel G-quadreplex single-stranded DNA aptamer, with ability to target *S. aureus* protein A.^93^ The aptamer acted as the biorecognition element to specifically target the *S. aureus* biofilm, where the gradual release of ampicillin led to the suppression of bacterial biofilm in bone tissue in a mouse model.

Another approach is to leverage electrostatic attraction interactions between nanocarriers and biofilms. Since EPS substances (polysaccharide skeleton, proteins, humic and uronic acids, and DNA) are all negatively charged, they can be targeted to positively charged nanocarriers^94^ This concept was applied by Pedraza et al. decorating the outer surface of MSNs with N-(2-aminoethyl)-3-aminopropyltrimethoxy-silane.^95^ The protonation of amine groups provided the MSNs with positive charges, which increased the affinity of the nanosystem for *S. aureus* biofilm and increased the antimicrobial effect of the antibiotic cargo. In the same line of research, MSNs decorated with polycationic dendrimers (G3) exhibited biofilm-targeting ability, which synergistically improved the antimicrobial efficacy of the antibiotic payload against *E. coli* biofilms.^61^

Novel design strategies of organically modified MSNs have been explored for a fast and accurate bacterial separation from the sampling matrix, which could be of importance to reduce diagnosis time and planning therapy.^96^ Thus, Zheng et al. were able to graft temperature- and pH-responsive polymers to the surface of MSNs for the separation and enrichment of bacteria.^97^ They used poly(*N*-isopropylacrylamide-*co*-glycidyl methacrylate to which boronic acid was grafted, so bacteria interacted with them through boronic ester bonds, and Gram-negative bacteria were captured. In a different approach, selective separation of bacteria over mammalian cells was carried out decorating MSNs with vancomycin.^73^ They found that vancomycin modified MSNs selectively bounded *S. aureus* thanks to the affinity of vancomycin to Gram-positive bacteria.

An interesting technique of bacteria separation from the sampling matrix relates to the magnetic properties of specially designed MSNs, which can be employed to coat magnetic NPs in a core–shell approach.^98^ The use of mesoporous silica shells enhances the colloidal stability of the magnetic NPs allowing the capture of bacteria at ultralow concentration.

MSNs have also been employed in the design of biosensors for detection of bacterial infection. Gu et al. designed MSNs with a chemiluminescence material on their surface and capped with DNA.^99^ Then, the DNA nuclease enzyme (analyte for bacterial detection) binds to the DNA present at the surface of the MSNs and triggers the release of the chemiluminescence molecule indicating the presence of bacteria. Different biosensors have been designed through this approach of modifying the surface of MSNs to improve the detection limits and sensitivity.^96^

In general, these have many different engineered MSNs strategies that have been designed to target bacteria, which represents a potent alternative for fighting bacterial infections. Whether in the planktonic state or associated in communities forming biofilms, delivering antimicrobials exclusively at the target site would avoid affecting healthy tissues and increase efficacy of the treatment. Table 1 collects some of the most relevant strategies described in the literature.^100^

**Table 1:** Most Relevant Engineered MSNs to Target Bacteria^100^

Nanocarrier	Drug Loaded	Targeting Ligand	Bacteria	ref.
MSNs-Antibody	Fluoresceine and Hoechst 33342 model drugs	FB11 antibody for lipopolysaccharide	*F. tularensis*	(67)
Sulfonated-Hyaluronic acid-MSNs	Vancomycin	Anti-*S. aureus* antibody	*S. aureus*	(68)
MSNs-Aptamer	Vancomycin	SA20 aptamer	*S. aureus*	(69)
MSNs-Lipidic bilayer shell	Gentamicin	Ubiquicin	*S. aureus*	(81)
MSNs-Lipidic layer	Colistin	LL-37 peptide	*P. aeruginosa*	(82)
MSNs-Perfluorophenylazide	Isoniazid	Trehalose	*M. smegmatis*	(70)
MSNs-Trehalose	Isoniazid	Trehalose	*M. smegmatis*	(71)
MSNs-Arginine	Ciprofloxacin	Arginine	*S. typhimurium*	(80)
MSNs-Folic acid	Ampicillin	Folic acid	*E. coli, S. aureus*	(72)
MSNs-Vancomycin	Vancomycin	Vancomycin	*S. aureus*	(73)
MSNs-Outer membrane vesicle	Rifampicin	Outer membrane vesicle	*E. coli*	(74)
MSNs-Poly-l-lysine	Vancomycin	Poly-l-lysine	*E. coli*	(64)
MSNs-Poly-l-lysine	Histidine kinase authophosphorylation inhibitors	Poly-l-lysine	*E. coli*	(65)
MSNs-Lysozyme	Kanamycin	Lysozyme	*E. coli*	(66)
MSNs-Imine dendrimer	Levofloxacin	Poly(propyleneimine dendrimer)	*E. coli*	(61)
MSNs-Aminosilane	Levofloxacin	Amino-silane	*S. aureus*	(95)
MSNs-Cationic-imine dendrimer	Levofloxacin	Poly(propyleneimine dendrimer)	*E. coli*	(62)
MSNs-Concanavalin A	Levofloxacin	Concanavalin A	*E. coli*	(91)
MSNs-Arabic Gum	Moxifloxacin	Arabic gum	*E. coli*	(92)

### 3.2 Stimuli-Responsive Organically Modified MSNs

MSNs exhibit a plethora of advantages as drug delivery systems against AMR, but it is necessary to incorporate organic or inorganic nanogates to block the pores and prevent premature antimicrobial cargo leakage before reaching the target. Stimuli-responsive organically modified MSNs bring up the possibility of loading, protecting, and carrying the payload to the target location, and then releasing in response to given stimuli. These smart drug delivery nanosystems have the advantage of improving the pharmacokinetics and biodistribution of antimicrobial drugs, increasing their effective bioavailability, reducing their dosing frequency, and enhancing antimicrobial efficiency against resistant bacterial infections or slowing down the rise of AMR.^101,102^

Either internal (i.e., endogenous) stimuli, such as particular biological signals characteristic of the infection microenvironment, or external (i.e., exogenous) remotely controlled stimuli have been investigated as release triggers of antimicrobial agents from organically modified MSNs. The following sections describe the more innovative and ground-breaking strategies reported to date to design these smart nanosystems.

#### 3.2.1 Internal Stimuli Sensitive Organically Modified MSNs

**Figure 8:**
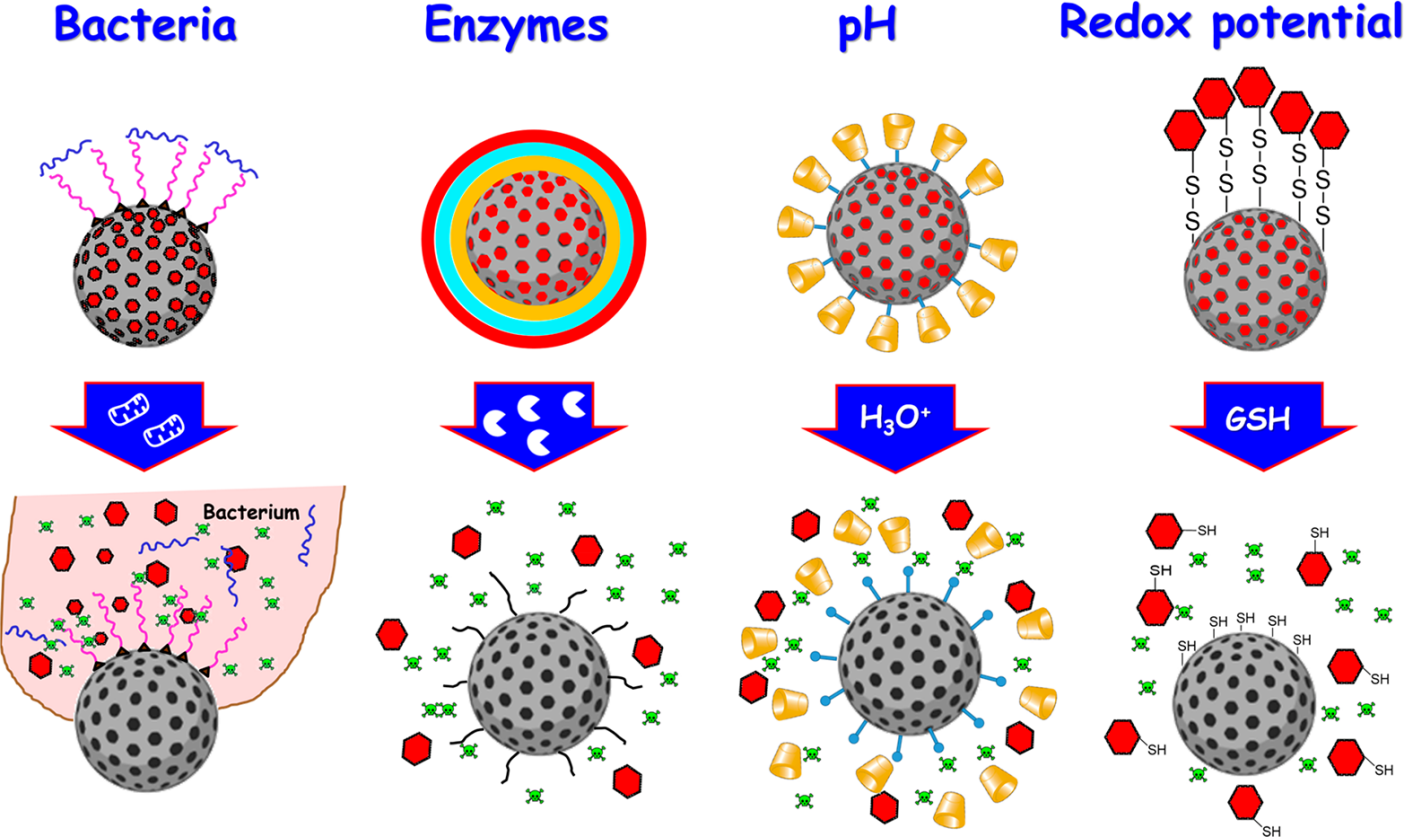
Schematic depiction of different internal stimuli used to trigger antimicrobials release from organic–inorganic hybrid MSNs against bacterial resistance.

Different internal stimuli that have been explored to trigger the release of antimicrobials from organically modified MSNs include the presence of bacteria, enzymes, pH, and redox potential (Figure 8).

##### 3.2.1.1 Presence of Bacteria

The pathogenic bacteria responsible for the infectious process itself can be used as a trigger for the release of antimicrobials from organically modified MSNs. Along this line, Mas et al. reported the capping of polycarboxylated-MSNs with cationic ε-poly-l-lysine (ε-pLys), through electrostatic interactions, to improve the antimicrobial effect of vancomycin against planktonic Gram– bacteria.^64^ In this research, the ε-pLys played a triple role, as a targeting, capping, and bacteria-sensitive agent. In the presence of the pathogen, the affinity of the negatively charged cell wall toward positively charged ε-pLys triggered pore opening and vancomycin release. Moreover, bacterial cell wall damage produced by ε-pLys aided the antibiotic penetration and avoided the emergence of bacterial resistance, which is quite common when the free antibiotic is administrated. An equivalent nanosystem was developed by Velikova et al.^65^ to increase the antimicrobial activity of histidine kinase autophosphorylation inhibitors. This nanosystem efficiently eradicated both Gram+ and Gram– planktonic bacteria while allowing the treatment on mammalian cells, as suggested by viability and immunotoxicity tests on zebrafish.

Alsaiari et al. developed innovative MSNs as dental nanofillers for bacterial detection and treatment.^66^ The nanofillers consisted in positively charged aminated MSNs were loaded with kanamycin and capped, through electrostatic interactions, with negatively charged gold nanocluster–lysozyme (AuNC@LYS) colloids. The presence of planktonic bacteria triggered the detachment of AuNC@Lys from MSNs, the quenching of the AuNC@Lys fluorescence, and the release of antibiotics.

The approaches described above lack specificity, which can be a disadvantage for sensing and treating infections produced by specific pathogens. Along this line, Kavruk et al. developed aptamer-gated MSNs for selective antibiotic delivery against *S. aureus* infections.^69^ Vancomycin-loaded MSNs were gated with the SA20 hp aptamer, which forms a hairpin locking structure. The binding of nanosystems to antigens present on the surface of *S. aureus* disrupted the hairpin structure of the aptamer and released the antibiotic cargo. In another research paper, Ruehle et al. modified the surface of antibiotic-loaded MSNs with a derivative of the O-antigen of the lipopolysaccharide (LPS) of *Franciscella tularensis* (*F. tularensis*) and then capped the mesopores with the FB11 antibody.^67^ In the presence of the target bacterium, the FB11 antibody effectively bonded with the native LPS on the outer membrane of *F. tularensis*. Interaction of the antibody with the antigen produced a pore opening and allowed the release of the antimicrobial payload. The excellent selectivity of this nanosystem reduced side effects and decreased the risk of resistance compared to the use of conventional broad-spectrum antibiotics.

##### 3.2.1.2 Enzymes

The design of smart enzyme-triggered antimicrobial drug delivery systems against bacterial infection is receiving growing attention.^103^ The presence of enzymes secreted by bacteria, such as lipase, hyaluronidase, protease, and antibiotic degrading enzymes in infected microenvironments can be used as efficient release triggers. For instance, Wu et al. developed a hyaluronidase-responsive biohybrid nanosystem consisting on amoxicillin-loaded MSNs coated by the layer-by-layer self-assembly method with lysozyme, hyaluronic acid, and 1,2-ethanediamine (EDA)-modified polyglycerol methacrylate (PGMA).^104^ In the nanosystem, the lysozyme and cationic PGMA derivative efficiently binds to the bacteria cell wall due to multivalent interactions, whereas hyaluronic acid operates as enzyme hyaluronidase-responsive nanogates for antibiotic release. The synergistic combination of the different building blocks in a unique nanosystem efficiently eradicated amoxicillin-resistant *S. aureus in vitro* and *in vivo* in a wound infected mouse model. Xu et al. engineered hyaluronidase-responsive antibiotic release MSNs to develop “on-demand” nanoplatforms for diagnosis and treatment of *S. aureus* infection in the bloodstream.^68^ For this purpose, magnetic MSNs were loaded with vancomycin, coated with a sulfonated-hyaluronic acid, and decorated with a *S. aureus* antibody. The nanosystem was deposited on a magnetic glassy carbon electrode. The specific antigen–antibody interaction between *S. aureus* in solution and the antibody on the electrode surface produced changes in the electrochemical signals, which allowed the precise detection of the amount of *S. aureus* in solution. The anticoagulant properties of this nanosystem allowed the prepared immunosensor to be applied in whole blood. The increase of the amount of *S. aureus* reaching the electrode increased levels of the secreted hyaluronidase, degrading the capping agent and releasing antibiotic to effectively kill *S. aureus*.

Secreted bacterial enzymes, including extracellular enzymes such as lipases,^105^ were proposed as endogenous stimuli to develop advanced responsive MSNs against intracellular infections. The novelty of these intelligent nanosystems was to coat MSNs with a liposomal shell and then conjugate a specific AMP, namely, (UBI)_29–41_ or LL-37.^81,82^ In these nanosystems, AMP was the targeting ligand toward pathogenic intracellular bacteria and the lipid shell of the pore capping agent to prevent antibiotics inactivation and premature release before reaching the site of action. The liposome bilayer is degraded by secreted lipase present in the in the local environment of intracellular bacteria, allowing the release of the antibiotic cargo for the efficient elimination of pathogens.

##### 3.2.1.3 pH

Bacterial infection produces a noticeable pH decrease in the local microenvironment through anaerobic fermentation, activated by hypoxia conditions, and inflammatory immune system responses. pH at the infection site can reach values as low as 5.5,^106^ which can be used to design pH-sensitive antimicrobial nanosystems against bacterial infection.

Some pH-responsive MSNs make use of pH-cleavable bonds or polymers that undergo pH-dependent conformational changes. For instance, Kuthati et al. decorated MSNs with silver-indole-3 acetic acid hydrazide (IAAH-Ag) complexes through a pH-cleavable hydrazone bond to evaluate the ability of this combination to eliminate pathogenic planktonic bacteria or biofilms.^107^ The pH-responsive complex showed a concentration-dependent inhibitory effect toward *E. coli* and *S. aureus* with improved inhibition toward the latter. The antibacterial actions produced by MSNs toward tested bacteria appear to be a complementary effect of their ability to decrease the amount of genomic DNA produced, the generation of reactive oxygen species, and their ability to enable movement through the complex biofilm structure even at 30 μg mL^–1^. In another research work, Yan et al. developed a pH-responsive hydrogel for detection and killing of bacteria.^108^ First, the external surface of vancomycin-loaded MSNs was decorated with fluorescein isothiocyanate (FITC). At this point, the NPs emitted strong green fluorescence in basic or neutral pH conditions, whereas the emission was reduced at acidic pH values because of the pH-sensitive property of FITC. Then, the pH-sensitive polymer poly(*N*-isopropylacrylamide-*co*-acrylic acid) was copolymerized with a derivative of rhodamine B, functionalized with a rhodamine-B-based derivative (RhBAM), and grafted onto MSNs. At neutral or basic pH RhBAM was present in the spirolactam form (no fluorescence), while at acidic pH values it changed to the open form and emitted strong red fluorescence. Organically modified MSNs were immobilized in an agarose matrix layer to detect and kill bacteria. Protons produced by bacteria not only caused the hydrogel to change color from green to red, but also triggered the release of antibiotics to inhibit the growth of *E. coli*.

A different approach was to develop pH-responsive nanosystems using pH-degradable capping elements. In this line, Duan et al.^109^ designed an innovative nanosystem for efficient treatment of MRSA infections, which is difficult due to the fact that β-lactam antibiotics can undergo enzymatic degradation and cannot penetrate deeply into biofilms. They developed metalcarbenicillin framework-coated MSNs as a codelivery system for β-lactam antibiotics and β-lactamase inhibitors. Carbenicillin, a β-lactam antibiotic, was used as a ligand for Fe^3+^ to generate a metalcarbenicillin framework that acted as pH-sensitive pore capping agents. This research showed that this nanosystem reached deeper penetration into biofilms and showed an inhibitory effect on MRSA biofilms both *in vitro* and *in vivo*. In another report, Chen et al. developed pH-responsive nanosystems by coating ampicillin-loaded MSNs with folic acid (targeting ligand) and calcium phosphate (CaP, pH-degradable capping agent) to inhibit antibiotic-resistant *S. aureus*.^72^ The nanosystem reduced the content of altered membrane proteins, bypassing the bacterial efflux pump system and killing resistant bacteria. The acidic pH degradation of CaP triggered ampicillin release, inhibiting bacterial growth *in vitro* and *in vivo*. In another report, Abdelbar et al. developed a pH-responsive nanosystem by coating levofloxacin-loaded MSNs with pH degradable polylactic acid nanoflowers.^110^ At neutral pH the nanoflowers created a compact capping layer on MSNs, whereas at acidic pH the capping shell was degraded, triggering antibiotic release. The antimicrobial efficacy of the nanosystem against planktonic *S. aureus* and *E. coli* was successfully demonstrated *in vitro*.

In order to reduce the risk of developing MDR due to antibiotic exposure, some authors developed pH-responsive multifunctional MSNs as codelivery systems of antimicrobial drugs and antimicrobial metal ions. For instance, Lu et al. loaded the antiseptic drug chlorhexidine into silver-decorated MSNs to evaluate the bactericidal effect against *S. aureus* and *E. coli*.^111^ The nanosystem was designed to simultaneously release chlorhexidine and Ag^+^ in a pH-responsive fashion, leading to the synergistically antibacterial effect against the Gram+ and Gram– tested bacteria. These nanoantiseptics exhibited good biocompatibility on normal cells at the efficient antibacterial doses. In another work, Kankala et al. developed a trio-constructs-based pH-responsive nanosystem for synergistic antibacterial treatment of MDR infections. Initially, tetracycline-loaded MSNs was impregnated with copper ions, establishing pH-responsive coordination interactions with the guest drug molecules.^112^ Then the resulting nanosystem was coated by an ultrasmall silver NPs-stabilized PEI layer. *In vitro* bioassays against MDR *E. coli* indicated that the release of silver ions improved antibacterial capacity by sensitizing the cell wall, which enhanced intracellular availability of the nanocarriers for pH-responsive release of antibiotic drug. Moreover, huge ROS levels produced by Cu species in the surface of MSNs allowed the eradication of MDR bacteria.

Antimicrobial therapy against intracellular infections can also take advantage of pH-responsive MSNs. Along this line, Clemens et al. developed pH-gated MSNs as isoniazid release systems to combat *M. tuberculosis* infection.^78^ To this aim, MSNs were equipped with pH-operated nanovalves based on beta-cyclodextrins (β-CDs), which were built by covalent grafting of molecular threads over the mesopores followed by the addition of bulky β-CDs that, at neutral pH, bind the threads and sterically block the pores. Acidic pH produces the protonation of molecular threads and decreases their binding affinity toward the β-CDs blocking caps, triggering opening of the nanovalves and allowing antibiotic release. The successful antibacterial effect of the pH-operated nanosystems was *in vitro* demonstrated against tuberculosis-infected human macrophages. Similar pH-operated nanomachines were employed as moxifloxacin release systems to eradicate *F. tularensis* infection in a mouse model of pneumonic tularemia.^113^ In another work, Hwang et al. innovated another approach to develop a prodrug nanoformulation by covalently grafting isoniazid to MSNs through hydrazone bonds.^114^*In vivo* evaluation in a mouse model of pulmonary tuberculosis demonstrated the pronounced efficiency of the nanoformulation compared to free administration of antibiotic.

##### 3.2.1.4 Redox Potential

The most reducing intracellular environment compared to the extracellular medium is due to the numerous redox pairs involved in many metabolic pathways.^115^ This is the case of the reduced/oxidized glutathione (GSH/GSSG) redox pair, which has been extensively exploited to develop redox-responsive MSNs for cancer treatment.^116^ More recently, different research teams have applied the acquired knowledge to design smart MSNs against bacterial resistance. Lee et al. designed a redox-responsive nanosystem to treat intracellular infections which was based on MSNs loaded with moxifloxacin and functionalized with disulfide snap-tops.^117^ First, MSNs were functionalized with (3-mercaptopropyl) trimethoxysilane and then reacted with adamantanethiol to form a disulfide bond. Following drug loading, β-CDs were added as blocking caps due to their ability to form inclusion complexes with adamantanethiol moieties. *In vitro*, this disulfide bond was cleaved in the reducing milieu inside the macrophages, allowing cargo release and inhibiting *F. tularensis*. In *in vivo* assays in a mouse model of lethal pneumonic tularemia, this nanosystem prevented premature death and significantly diminished the presence of the pathogen in the spleen, lung, and liver.

Overexpression of ROS in infected microenvironments provides the opportunity to design nanoformulations sensitive to ROS.^118^ Within this framework, Li et al. designed an ROS-responsive nanosystem by loading aminated-MSNs with vancomycin and subsequently grafting with a thioketal functionalized methoxy poly(ethylene glycol) gatekeepers.^119^ The interaction with the ROS in the microenvironment caused the thioketal linker and the polymer coating to rupture, allowing the release of the antibiotic cargo. *In vitro* assays against *S. aureus* proved the enhanced antimicrobial effect of the nanosystem compared to that of the free antibiotic, which was attributed to strong influence on the bacterial membrane’s disintegration. A satisfactory antibacterial effect was also observed in a rat-infected skin wound model.

#### 3.2.2 External Stimuli Organically Modified MSNs

**Figure 9:**
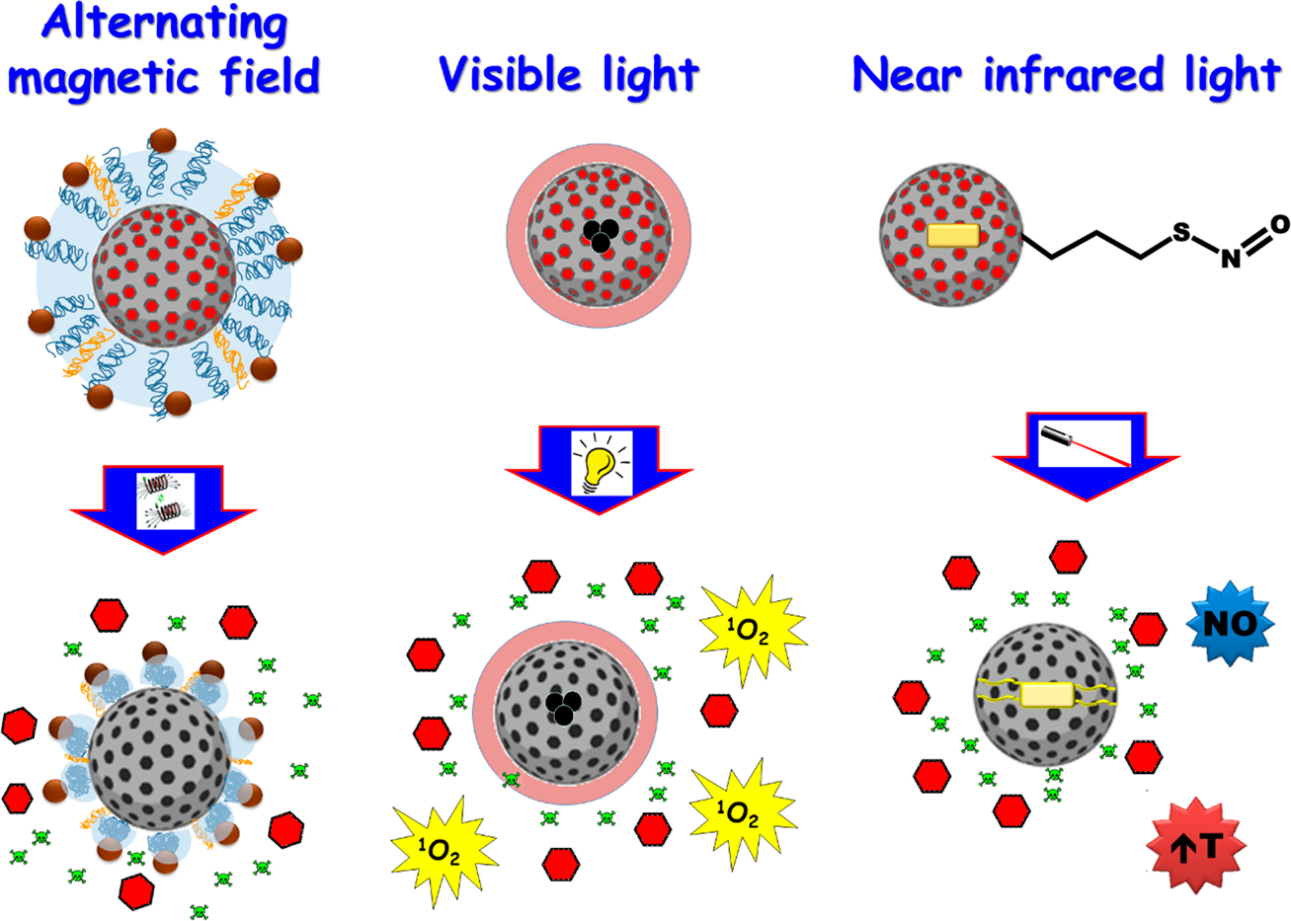
Schematic representation of different external stimuli used to trigger antimicrobials release from organically modified MSNs for bacterial infection treatment.

The main external stimuli used to trigger antimicrobials delivery from organically modified MSNs comprise alternating magnetic field, visible light, and near-infrared light (Figure 9).

##### 3.2.2.1 Alternating Magnetic Field

Magnetic fields own the best penetration of tissue of the three external stimuli discussed in this article. Superparamagnetic iron oxide nanoparticles (SPIONs) generate heat in the presence of an alternating magnetic field (AMF). Thus, SPIONs can be incorporated into antimicrobial-loaded MSNs coated with thermosensitive nanogates to trigger pore uncapping upon application of an AMF. Thus, Yu et al. engineered a sophisticated AMF-responsive nanoplatform to simultaneously deliver multiple drugs.^120^ In such a work, core–shell SPIONs@MSNs loaded with ofloxacin were coassembled with large-pore MSNs loaded with the AMP melittin. This smart nanosystem was AMF-sensitive and also responded to pathogen bacteria, codelivering melittin and ofloxacin to synergistically kill MDR *P. aeruginosa* bacteria. Moreover, the nanosystem accomplished highly efficient targeting with pathogenic biofilms under AMF and pathogen stimuli. The supramolecular dual coassembly of drug-loaded heterogeneous MSNs efficiently eradicated *in vivo* pathogenic biofilms from implants and prevented host tissue damage and inflammation. In another recent work, Álvarez et al. developed an AMF-responsive antibiotic delivery nanosystem against *E. coli* bacterial biofilms.^121^ MSNs were decorated with two different polymers: polyethylene glycol (PEG), to increase colloidal stability; and a poly-*N*-isopropylacrylamide (PNIPAM) derivative, as the thermosensitive element that undergoes a conformational change (linear-to-globular) at a temperature above 40–43 °C. Then, the polymer-coated MSNs were decorated with magnetite SPIONs and loaded with levofloxacin following a temperature-controlled process. In this nanosystem, SPIONs played a triple role: (i) behaving as hot spots, causing the shrinkage of PNIPAM chains upon application of an AMF and triggering cargo release; (ii) favoring biofilm-eradication by hyperthermia due to the intimate contact between SPIONs and biofilm; and (iii) exerting the antimicrobial effect by themselves due to their chemical nature. In vitro assays against *E. coli* biofilms showed efficient antimicrobial behavior in the presence of an AMF, significantly decreasing the bacteria viability.

##### 3.2.2.2 Visible Light

Visible light is receiving growing attention due to the opportunity to synergistically combine photoinduced antimicrobials release and phototherapy against bacterial resistance. Kuthati et al. developed a smart antimicrobial nanosystem, termed as a trio-nanosystem, against antibiotic resistant Gram– bacteria.^122^ The trio-nanosystem consisted of MSNs loaded with curcumin, impregnated with Cu^2+^ ions and decorated with Ag NPs. The illumination of the trio-nanosystem with blue-LED light produced an effective photodynamic inactivation effect against antibiotic resistant *E. coli*. In this system, curcumin can produce high amounts of ROS under light irradiation, which can additionally increase the silver ion release kinetics for antibacterial effect. Moreover, the positive charged modified surfaces of Cu-MSN favored an antimicrobial response via electrostatic attracting interactions with the negatively charged bacteria cell wall. In another work, Liu et al. fabricated multifunctional nanoplatforms based in organically modified MSNs for drug delivery and imaging-guided chemo/photodynamic synergistic therapy.^123^ To build the multicomponent nanosystem, carbon dots (C-dots) and a photosensitizer, rose bengal (RB), were embedded in core/shell structured MSNs. Finally, ampicillin was loaded into the mesopores. In this system, C-dots can serve as a fluorescence probe to achieve cell fluorescence imaging and RB can generate singlet oxygen to perform photodynamic therapy (PDT). *In vitro* assays in *E. coli* cultures showed that upon green light illumination, the ampicillin-free nanosystem significantly reduced the number colony forming units (CFUs) compared to the control (no light irradiation), evidencing the generation of singlet oxygen. On the other hand, the antibiotic-loaded nanosystem produced total *E. coli* growth inhibition under green light irradiation, proving the enhanced synergetic bacterial growth inhibition effect of the whole nanosystem.

##### 3.2.2.3 Near-Infrared Light

Near-infrared (NIR) laser light irradiations can be used to combine trigger drug delivery from light-responsive MSNs with photothermal therapy (PTT). PTT refers to the efficient conversion of light (most often in NIR wavelengths) in localized heating, mediating the strong absorption of certain metallic nanoparticles and nanomaterials.^124−126^ Antibacterial PTT has attracted intensive attention due to its high specificity and capacity to induce bacterial cell death and biofilm destruction.^127^ Nevertheless, the nonlocalized heat may damage healthy tissues, which become a great opportunity for MSNs based nanocarriers. In this line, García et al. developed a new nanoassembly with photothermal and anticrobial capabilities to combat *S. aureus* biofilms.^128^ In such nanosystem, gold nanorods (AuNR) served as the cores, and MSNs acted as the shell to form core–shell structures named AuNR@MSNs. Then, the AuNR@MSNs was functionalized with the nitrosothiol group, which acted as an NO donor, and the antibiotic levofloxacin was loaded into the mesopores. Upon 808 nm light illumination, the temperature of the nanosystem produced a photothermal effect and triggered the release of NO and levofloxacin, which led to a *S*. *aureus* biofilm reduction of 90%.

As it has been mentioned above, MSNs can also be designed to load, protect, and transport antibacterial agents to the site of interest, and once there, release the payload only upon the exposure of certain triggers, as it has been above-mentioned. Table 2 collects some of the most interesting organically modified MSNs that release their antimicrobial cargo in response to certain stimuli.

**Table 2:** Most Relevant Engineered Stimuli-Responsive MSNs to Treat Infection

Nanocarrier	Drug Loaded	Stimulus	Bacteria	Ref.
MSNs-Poly-l-lysine	Vancomycin	Presence of bacteria	*E. coli*	(65)
			*S. typhi*	
			*E. carotovora*	
MSNs-Poly-l-lysine	Histidine kinase autophosphorylation inhibitors	Presence of bacteria	*E. coli*	(65)
			*S. marcescens*	
MSNs-Gold nanocluster lysozyme	Kenamycin	Presence of bacteria	*E. coli*	(66)
MSNs-Aptamer	Vancomycin	Presence of bacteria	*S. aureus*	(69)
			*S. epidermis*	
MSNs-Antibody	Fluorescein as model molecule	Presence of bacteria	*F. tularensis*	(75)
MSNs-Layer by layer coated	Amoxicilin	Bacterial toxins	*S. aureus*	(104)
MSNs-Sulfonated hyaluronic acid	Vancomycin	Bacterials toxins	*S. aureus*	(68)
MSNs-Lipid shell bilayer	Colistin	Enzymes secreted by bacteria	*Pseudomonas aeruginosa*	(81)
				(82)
MSNs-Hydrazone	Silver complex as a model drug	pH	*E. coli*	(107)
			*S. aureus*	
			*B. subtilis*	
			*S. epidermis*	
MSNs-Poly(*N*-isopropylacrylamide-*co*-acrylic acid)	Vancomycin	pH	*E. coli*	(108)
MSNs-Carbenicillin	Lactam antibiotic and lactamase inhibitors	pH	*S. aureus*	(109)
MSNs-Folic acid and degradable calcium phosphate	Ampiallin	pH	*E. coli*	(72)
			*S. aureus*	
MSNs-Poly(lactic acid) nanoflowers	Levofloxacine	pH	*E. coli*	(110)
			*S. aureus*	
Nanosilver-decorated MSNs functionalized with carboxylate	Chlorhexidine with Ag^+^ ions	pH	*E. coli*	(111)
			*S. aureus*	
MSNs-Beta-cyclodextrin nanovalves	Isoniazid	pH	*M. tuberculosis*	(78)
				(114)
MSNs-Beta-cyclodextrin nanovalves	Moxifloxacin	pH	*F. tularensis*	(113)
MSNs-Disulfide bonds	Moxifloxacin	Redox	*F. tularensis*	(117)
MSNs-GSH degradable	Gentamycin with Ag^+^ ions	Redox	*E. coli*	(129)
			*P. aeruginosa*	
			*S. aureus*	
			*E. faecalis*	
Disulfide-bridged MSNs with nanosilver and carboxylate	Chlorhexidine with Ag^+^ ions	Redox and pH	*S. mutans*	(130)
MSNs-Thioketal	Vancomycin	Reactive Oxygen Species	*S. aureus*	(119)
MSNs-Layer by layer supramolecular nanoassembly	Amoxicillin	Adamantaneamine	*E. coli*	(131)
			*S. aureus*	
Iron oxide-MSNs core shell with MSNs-poly(*N*-isopropylacrylamide)	Lysozyme	Temperature	*Bacillus cereus*	(132)
			*Micrococus luteus*	
MSNs with silver nanoparticles	Curcumin	Light	*E. coli*	(122)
MSNs-C dots and rose Bengal	Ampicillin	Light	*E. coli*	(123)
Core–shell gold MSNs	Levofloxacin	Near Infrared	*S. aureus*	(128)
MSNs incorporating superparamagnetic iron oxide NPs	Melittin and ofloxacin	Alternating Magnetic Field	*P. aeruginosa*	(120)
MSNs-Poly(*N*-isopropylacrylamide) decorated with superparamagnetic iron oxide NPs	Levofloxacin	Alternating Magnetic Field and Temperature	*E. coli*	(121)

The present review has demonstrated the potential of MSNs to treat infectious diseases. However, there are several different challenges that remain to be explored before accomplishing their translation to the clinic. Most of the studies involving NPs in general, and MSNs, to potentially treat bacterial infections have been carried out under *in vitro* conditions, with few systematic in vivo studies. There is a clear need of exploring these formulations *in vivo* to be able to advance the preclinical stage toward clinical trials.

Additionally, there is a need to deeply understand how MSNs combat biofilms, because up to date there are few studies on the antibiofilm mechanics of MSNs and nanomedicines in general. There are also several challenges associated with MSNs that should be addressed before clinical translation, such as blood circulation stability, clearance mechanisms, and potential metabolic effects to the host.

## 4 Conclusion and Outlook

Some bacteria present in the biofilm are particularly pathogenic due to their resistance to antimicrobial treatment, which forces an increase of the dose of drugs to be administered up to 1000 times higher than that needed for their planktonic counterparts. In this sense, the use of MSNs brings some advantages in combating biofilm infections and, in general, drug resistant bacteria. Some of these advantages come from the fact that they can act on all stages of bacterial biofilm formation and diffusion. In this regard, MSNs can be designed to specifically target the bacteria present on the biofilms, as it has been mentioned above, transporting therapeutic agents capable of destroying extracellular polymeric substances and, therefore, enlarging the biofilm permeability to antimicrobial therapeutic agents. This is of capital importance because the extracellular polymeric substances matrix normally acts as a physicochemical barrier to protect the bacteria limiting the penetration of antibiotics. Another advantage of MSNs is their capacity of protecting the transported antimicrobial substances from enzymatic inactivation and from the potential binding to DNA and polysaccharides produced by biofilms. In addition to penetrating the biofilm and destroying the EPS barrier, MSNs can transport a great amount of many different therapeutic agents and/or biomolecules with antimicrobial activity. In this sense, one of the best qualities of MSNs is their capacity to multisite transport different types of drugs against both bacteria and EPS. Besides, the release of the payload can be controlled, delaying the release rate and, therefore, prolonging the bacterial-killing time window of the different antimicrobial substances, which allows a better antibiofilm effect. A very interesting feature of MSNs that has been mentioned throughout this review is their capacity to internalize into bacteria cells, which ensures the release of the antimicrobial agents into the right place without affecting the rest of healthy cells and, therefore, avoiding side effects. Last, but not least, engineering MSNs allows the design of smart and multifunctional nanocarriers, releasing a cocktail of therapeutic agents and antibiofilm substances where they are required when they might be needed.

On the other hand, MSNs present some disadvantages to treat drug resistant bacterial infections, which are responsible for their absence of the clinical arsenal to fight infections. First, MSNs are still in the preclinical stage. Although there are other type of silica NPs in clinical trials, such as Cornell dots, MSNs are still far from being translated to the clinic. Additionally, their batch-to-batch variability makes the production reproducibility a challenge difficult to reach. This lack of reproducibility necessarily affects their manufacturing scaling-up and, therefore, the access to the biomedical market. From a more technical point of view, MSNs have been explored to combat drug resistant bacteria mainly in *in vitro* scenarios. There is a need for more realistic *in vivo* models to test MSNs because the properties and behaviors of these MSNs in the body are uncontrollable. For example, the stability of MSNs within the body is a challenge that needs to be taken into consideration through a careful design of their surface to maintain the dispersion of MSNs in the biological environment. Finally, considering the importance of safety of all nanomaterials, there is a need to evaluate the behavior of MSNs engineered to fight infections in relevant *in vivo* models to ensure a good biodistribution and avoid any potential toxicity because of the nanocarriers design.

In this perspective article we have outlined the recent advances in the design and development of organically modified MSNs that improve the administration of antimicrobial drug and treatments for bacterial infection. The enormous potential of these nanosystems to circumvent bacterial resistance mechanisms is due to their multifunctionality derived from the assembly of different inorganic and organic building blocks for therapeutic purposes. Various studies have shown the potential of multifunctional MSNs to improve targeting, control drug release performance, and improve antibacterial activity, mainly against antibiotic-resistant planktonic bacteria, intracellular bacterial strains, and bacterial biofilms.

In addition to releasing antibiotics, some multifunctional MSNs also incorporate inorganic metal ions, showing a more prominent antibacterial effect due to the synergistic combination and codelivery of antimicrobial cargoes. Moreover, the possibility of modifying MSNs with different stimuli-responsive entities to prevent cargo leakage before reaching the target significantly improves the therapeutic outcome. In addition, for the in situ diagnosis and treatment of pathogenic bacterial infections, the possibility of establishing funcionalized MSNs with diagnostic functions, targeting capacity and triggered release of antimicrobial cargoes in response to internal or external stimuli is being explored. Nonetheless, albeit multifunctional MSNs being promising candidates as nanotheranostics agents in antibacterial infection therapy, this is still an emerging research field.

Although the great potential of these nanosystems against bacterial infection is evident, they have not yet translated into the clinic. More studies are needed to overcome the challenges associated with their multiple components; nanoformulation optimization; production reproducibility; manufacturing scaling-up; and cost-effective development of organically modified MSNs to obtain regulatory agencies approval. In addition, *in vivo* assays on large animal infection models, such as mini-pigs, sheep, or goats, are required to mimic the human response as much as possible and assess toxicity, stability, pharmacokinetics, and *in vivo* biodistribution of the nanosystems.

The present review has demonstrated the potential of MSNs to treat infectious diseases. However, there are several different challenges that remain to be explored before accomplishing their translation to the clinic. Most of the studies involving nanoparticles in general, and MSNs in particular, to potentially treat bacterial infections have been carried out under *in vitro* conditions, with few systematic *in vivo* studies. There is a clear need of exploring these formulations in vivo to be able to advance the preclinical stage toward clinical trials.

Additionally, there is a need to deeply understand how MSNs combat biofilms, because up to date there are few studies on the antibiofilm mechanics of MSNs and nanomedicines in general. There are also several challenges associated with MSNs that should be addressed before clinical translation, such as blood circulation stability, clearance mechanisms, and potential metabolic effects to the host.

Today, the development and antibacterial applications of multifunctional organically modified MSNs are still in their infancy. This challenging scenario calls for the effort of multidisciplinary teams, where physicians, scientists, and technicians work together to promote industrial transfer and clinical translation of this new generation of nanoformulations to combat bacterial resistant infections.
